# Military service and alcohol use: a systematic narrative review

**DOI:** 10.1093/occmed/kqac045

**Published:** 2022-06-08

**Authors:** A K Osborne, G Wilson-Menzfeld, G McGill, M D Kiernan

**Affiliations:** Northern Hub for Veterans and Military Families Research, Department of Nursing, Midwifery and Health, Faculty of Health and Life Sciences, Northumbria University, Newcastle-Upon-Tyne, UK; Northern Hub for Veterans and Military Families Research, Department of Nursing, Midwifery and Health, Faculty of Health and Life Sciences, Northumbria University, Newcastle-Upon-Tyne, UK; Northern Hub for Veterans and Military Families Research, Department of Nursing, Midwifery and Health, Faculty of Health and Life Sciences, Northumbria University, Newcastle-Upon-Tyne, UK; Northern Hub for Veterans and Military Families Research, Department of Nursing, Midwifery and Health, Faculty of Health and Life Sciences, Northumbria University, Newcastle-Upon-Tyne, UK

**Keywords:** Alcohol drinking, military personnel, military health, review

## Abstract

**Background:**

Despite research highlighting the role of alcohol in military life, specifically in relation to mental health and certain combat experiences, there is no synthesised evidence looking at the relationship between military service and alcohol use.

**Aims:**

To synthesize and examine evidence exploring the relationship between military service and alcohol use.

**Methods:**

Six databases were examined across a 10-year period. Papers were included if they involved a military population and focused on alcohol use. From 4046 papers identified, 29 papers were included in the review.

**Results:**

Military characteristics and experience were linked to high levels of alcohol use across military populations. Societal and cultural factors also played a role in alcohol use in military populations. Predatory behaviour of alcohol establishments, pressures to conform, an acceptance of alcohol use, and the role of religious services and military affiliated social networks were all considered. Excessive drinking impacted physical and mental health. Those diagnosed with PTSD and associated symptoms appeared to have greater alcohol use.

**Conclusions:**

This review identified certain characteristics and experiences of military service that are associated with higher levels of alcohol use. It is important to identify risk factors for alcohol misuse to develop appropriate policy, targeting prevention.

**Key learning points**

What is already known about this subject

- Historically alcohol has had an integral role in military life and has been seen as an acceptable behaviour in social bonding and comradeship.
- Alcohol misuse in military populations has been associated with a negative impact on social, physical, and psychological health.
- Literature reviews to date have focussed on comorbidity of PTSD and alcohol misuse, wider mental health and Gulf and Iraq/Afghanistan war veterans, no systematic reviews of literature have considered the wider experiences of alcohol use and military service.

What this study adds

- Military-specific traits and experiences such as, service type, rank and deployment status are linked to higher levels of alcohol use in military populations across multiple countries.
- There appears to be an over-reliance on self-report questionnaires for the assessment of alcohol use in a military population focussing on symptom severity, with a paucity of research considering personal experiences and meanings ascribed to alcohol use.
- The systematic narrative review has brought together a body of knowledge on alcohol use in a military population and the synthesis of this literature provides an evidence base to help inform future policy.

What impact this may have on practice, policy or procedure

- There are specific characteristics strongly associated with military service that impact alcohol use, it is important to identify these ‘risk factors’ to mitigate the impact on operational effectiveness and workplace cohesion by developing appropriate and targeted prevention policies.
- Mental ill health and harmful levels of alcohol use in military personnel co-exist and more specifically, this creates internal stigma making this population particularly reticent to seek help for both alcohol and mental health problems and therefore harder to identify.
- The systematic narrative review has highlighted a lack of consistency in the tools and measures used to assess alcohol use in a military population and suggests a need for a consensus of assessment measures in practice and wider research.

## Introduction

Alcohol has played a prevalent, historic role in military life, where, internationally, it has been used as a means of mediating stress, both in theatre and in the aftermath of battle [1]. Used in social bonding and comradeship [1], drinking has become a common and accepted behaviour in military culture, surpassing alcohol use in the general population [2, 3]. Beliefs on acceptable drinking norms can be influenced and reinforced when exposed to the military social environment [4].

The Motivational Model of Alcohol Use indicates consumption of alcohol may be used to cope, and to ‘regulate the quality of their emotional experience’ [5 p. 990]. These reasons for alcohol misuse have also been evidenced in serving military and veteran populations [4]. However, regardless of the potential advantages of alcohol consumption socially, especially in enhancing positive emotional experiences, problems develop when alcohol is misused. For military service members, exposed to highly stressful situations, behaviour around long-term alcohol use can be affected by the accepted social norms around higher levels of alcohol use for recreation and coping [4].

Research has suggested that alcohol may serve as a coping mechanism after traumatic events, where deployment has been associated with increased rates of alcohol use or problem drinking [6]. Drinking to excess may have a negative impact on mental and physical health [7], functional impairment [8], troop readiness [9], suicidal ideation [10] and the perpetrator in military sexual assaults [11]. Furthermore, the UK Armed Forces have expressed concerns that ‘excessive drinking can undermine operational effectiveness, leave soldiers unfit for duty, and damage trust and respect within the team’ [12 p. 12].

Alcohol use and military service are of great importance and a public health issue. However, there are no systematic reviews of literature that focuses on the wider, overall experiences of alcohol use and military service. Existing systematic review evidence stresses the important role of alcohol in military life, particularly focussing on the comorbidity of PTSD and alcohol misuse [13], Gulf and Iraq/Afghanistan war veterans [14], or as part of systematic reviews with a wider mental health focus [15]. Therefore, this study employs a systematic narrative review that aims to explore the relationship between military service and alcohol use.

## Methods

To appraise evidence from multiple sources including qualitative and quantitative research, and to ensure an inclusive systematic search without bias, a systematic narrative review strategy was employed [16]. Suitable databases were searched, identifying published peer-reviewed evidence (Table 1).

**Table 1.** Systematic search strategy

Source	ASSIA CINAHL PsycARTICLES PubMed central Science direct Web of science
Search field	Title, abstract, keywords
Language	English only
Exclusion	Non-English language Papers with veterans in their sample Papers looking at interventions or the use/development of services Papers that assessed/evaluated treatment for alcohol problems Papers that do not consider military service of the participants
Year of publication	All papers published between January 2001 and March 2021

Research papers on alcohol use with a military sample published prior to March 2021 were considered. Since the Global War on Terrorism began in 2001, there has been an increase in combat deployments of military service personnel from many nations across the globe. During this period, warfare has evolved, and the world has seen a more complex form of modern warfare, adapting and modernising to become more technologically advanced. This has also resulted in a change in the nature of deployments, impacting the role of military service in the lives of serving personnel. Deployments are dangerous and stressful for military personnel and combat stress, specifically experienced in Iraq and Afghanistan, has been associated with alcohol misuse [14]. Consequently, only papers published after 2001 have been considered in this review to ensure an accurate representation of the role military service has on alcohol misuse.

The exclusion criteria included papers that were unavailable in English, focussed on treatments or interventions for alcohol problems, or the sample included veterans. Papers on substance use were included, only if they reported alcohol use. The PICO framework was used to develop the search terms (Table 2). Truncation and wildcard search strategies were utilised.

**Table 2.** PICO framework to develop search terms

*P*	Patient or population	‘Veteran*’ OR, ‘Ex-Service*’ OR, ‘Soldier*’ OR, ‘Airm?n’ OR, ‘Sailor*’ OR ‘Recruit*’
*I*	Intervention	‘Military’ OR, ‘Armed Forces’ OR, ‘Army’ OR, ‘Navy’ OR, ‘RN’ OR, ‘Air Force’ OR, ‘RAF’ OR, ‘Marines’ OR, ‘Reserve*’ OR, ‘Home Guard’ OR ‘National Guard’
*C*	Comparison (if applicable)	Not applicable
*O*	Outcome	‘Alcohol*’ OR, ‘alcohol misuse’ OR, ‘alcohol use’ OR, ‘alcohol abuse’, OR ‘alcohol dependent’ OR, ‘drink*’, OR ‘Substance misuse’ OR, ‘substance abuse’ OR, ‘substance use’ OR, ‘Mental health’

**Figure 1.**
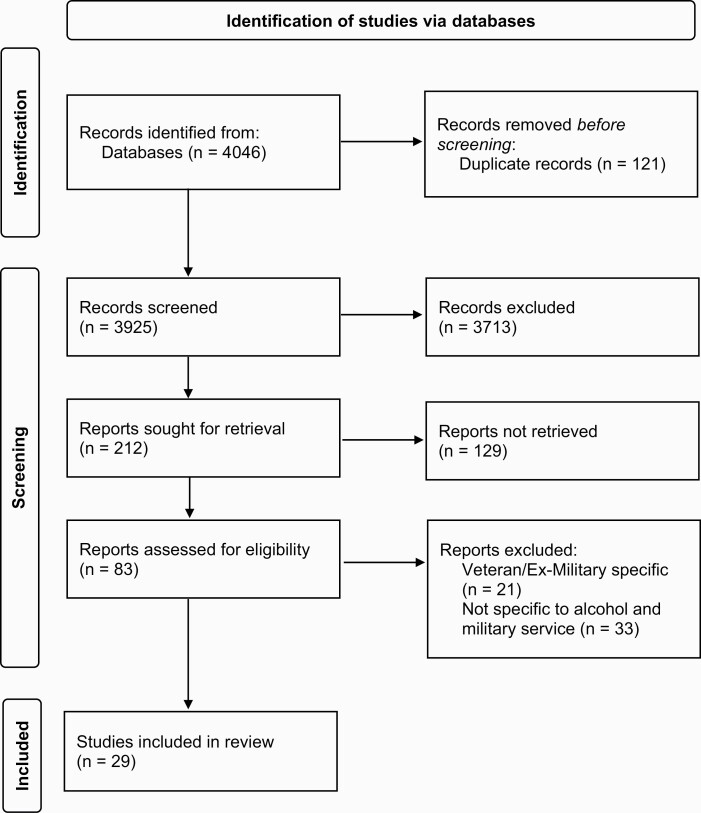
PRISMA diagram of papers returned during systematic search.

Veterans and ex-service were included as search terms to ensure the maximum number of papers were returned. However, only papers with a serving military population were included in the review. The ex-service military population was excluded because of additional, non-military specific, factors that exist that may impact alcohol use such as their experience of transition out of the military. Following the search of the databases, 4046 papers were returned (Figure 1).

A full-text search was carried out on 83 papers to determine the suitability for inclusion in the review. To appraise the quality of papers included in the review, the Critical Appraisal Skill Programme [17] tool was consulted. Fifty-four papers were excluded due to their focus on a veteran population or alcohol use not specific to military service. No further papers were identified through reference and citation searches. Consequently, 29 papers were included in this review.

Thematic analysis was used to analyse the papers and generate themes. The six steps of Braun and Clarke [18] were followed: familiarization with the data, generation of initial codes, searching for themes, reviewing themes, defining and naming themes, and producing the report.

## Results

Twenty-nine papers were included in this review (Table 3). There were 22 studies represented across all 29 papers. Studies with multiple papers in this review included: the Health and Wellbeing of the UK Armed Forces Cohort Study [2, 8, 39]; Ohio National Guard (OHARNG) Mental Health Initiative [34, 36]; Department of Defence Health Related Behaviours study [28, 35] and the Prevalence, Incidence and Determinant of PTSD and Other Mental Disorders (PID-PTSD^+3^) study [40–42].

**Table 3.** Characteristics of papers in review (*N* = 29)

Authors	Aim	Method	Sample size	Country of sample	Sample gender	Sample enlistment type
Besse *et al.* [6]	To understand the context of bars and restaurants in communities near military installations in relation to alcohol consumption	Qualitative	29	US	Mixed	Active duty
Browne *et al.* [19]	To explore the association between heavy drinking and military deployment factors	Quantitative	3578	UK	Male	Regular
Campbell-Sills *et al.* [20]	To identify prospective risk factors for post-deployment heavy drinking and alcohol or substance misuse disorder	Quantitative	4645	US	Mixed	N/S
Cerdá, *et al.* [21]	To explore the role of civilian stressors on alcohol use disorders	Quantitative	2616	US	N/S	National Guard
Cheng *et al.* [22]	To examine alcohol consumption patterns and related risk and protective factors	Quantitative	568	Angola	N/S	N/S
Dretsch *et al.* [23]	To examine the prevalence of the endorsed behavioural health problems and protective factors within a large sample of Special Operation Forces personnel	Quantitative	16 284	US	Mixed	Active Duty – Special Operations
Fadum *et al.* [24]	To examine self-reported physical and mental health in Norwegian military women and compare to active duty military men and civilian women	Quantitative	10 249	Norway	Mixed	Active Duty
Fear *et al.* [2]	To examine patterns of drinking in the UK Armed Forces, how they vary according to gender and other demographics, and to compare with the general population	Quantitative	8686	UK	Mixed	Regular
Ferrier-Auerbach*et al.* [25]	To examine relative contributions of known predisposing factors in a high-risk sample of Army National Guard soldiers	Quantitative	515	US	Mixed	National Guard
Goodell *et al.* [26]	To describe characteristics of soldiers’ social networks in association with soldier alcohol use problems	Quantitative	353	US	Male	National Guard and Reserves
Henderson *et al.*[27]	To compare alcohol use in the Royal Navy with a civilian population	Quantitative	1333	UK	Male	N/S
Herberman Mash *et al.* [28]	To examine the associations among drinking motives, alcohol use, PTSD, depression, and suicidality in US Army soldiers	Quantitative	3813	US	Mixed	Active Duty
Hooper *et al.* [29]	To investigate the association between traumatic exposure and substance use in a military sample, prospectively	Quantitative	941	UK	Mixed	N/S
Ijomanta and Lasebikan [30]	To determine the lifetime and 12 months prevalence of alcohol use and use disorders among a military population in Nigeria	Quantitative	223	Nigeria	Mixed	Regular
Kehle *et al.* [31]	To examine the associations between personality, PTSD symptoms and post-deployment alcohol use disorders among a group of deployed National Guard soldiers	Quantitative	348	US	Mixed	National Guard
Kline, *et al.* [32]	To examine the causal relationship of alcohol use, PTSD, and combat exposure	Quantitative	922	US	Mixed	National Guard
Larson *et al.* [33]	To identify the prevalence of self-report alcohol and psychological health problems immediately post-deployment	Quantitative	643 205	US	Mixed	All military personnel
Marshall *et al.* [34]	To examine whether pre-existing or coincident depression and PTSD predict new onset peri-/post-deployment alcohol abuse	Quantitative	963	US	Mixed	National Guard
Mattiko *et al.* [35]	To examine alcohol use patterns, drinking levels and self-reported negative outcomes	Quantitative	28 546	US	Mixed	Active Duty
Orr *et al.* [36]	To investigate the associations between deployment characteristics and alcohol use before and after incident	Quantitative	963	US	Mixed	National Guard
Rona *et al.* [8]	To consider the role of alcohol misuse in functional impairment in the military	Quantitative	8585	UK	Mixed	Regular
Russell *et al.* [37]	To examine the impact of combat experiences on alcohol use and misuse	Quantitative	263	US	Mixed	National Guard
Skipper *et al.* [38]	To examine the association between military combat experiences and post-deployment drinking in high-risk populations	Quantitative	1323	US	Mixed	Active Duty – Special Operations
Thandi *et al.* [39]	To investigate the impact of life events and changes in mental health status on AUDIT scores over time	Quantitative	5239	UK	Mixed	Regular
Trautmann *et al.* [40]	To investigate the prevalence of substance use and substance use disorders and the relationship between substance use and mental disorders	Quantitative	2372	Germany	Mixed	All serving personnel
Trautmann *et al.*[41]	To investigate diverse predictors and correlates of daily alcohol use following military deployment	Quantitative	358	Germany	Male	All serving personnel
Trautmann *et al.* [42]	To investigate the effect of internalizing disorders on the relationship between stress exposure alcohol use disorders and nicotine dependence in deployed military personnel	Quantitative	358	Germany	Male	All serving personnel
Vest *et al.* [43]	To explore cross-spouse effect of deployment and combat exposure on alcohol misuse in Reserve soldiers	Quantitative	248	US	Male	National Guard and Reserves
Wilk *et al.* [44]	To examine the association of specific types of combat experiences with a positive screen for alcohol misuse	Quantitative	1120	US	Mixed	N/S

AUDIT, Alcohol Use Disorder Identification Test; PTSD, Post Traumatic Stress Disorder; N/S, not specified.

Seventeen papers had samples from the US [6, 20, 21, 23, 25, 26, 28, 31–38, 43, 44], six from the UK [2, 8, 19, 27, 29, 39], three from Germany [40–42], one from Norway [24], one from Nigeria [30] and one from Angola [22]. Six papers looked at alcohol in the military for male personnel only [19, 26, 27, 41–43], whereas 21 papers considered male and female personnel [2, 6, 8, 20, 23–25, 28–40, 44]. Two papers did not specify the gender of their sample [21, 22].

Eleven papers considered Active Duty/Regular personnel [2, 6, 8, 19, 24, 28, 30, 35, 39] including two looking at special operations [23, 38]. Ten papers considered National Guard/Reserve personnel [21, 25, 26, 31, 32, 34, 36, 37, 43] and four papers considered all serving personnel [33, 40–42]. Five papers did not specify the enlistment type of the military personnel in their sample [20, 22, 27, 29, 44]

Of the 29 papers, 28 were quantitative [2, 8, 19–44] and one paper was qualitative [6].

Four themes were identified in the literature: Military Characteristics and Alcohol Use, Consequences of Deployment on Alcohol Use, Implication of Mental Health on Alcohol Use and The Role of Cultural and Social Factors on Alcohol Use.

Fifteen papers considered military characteristics associated with alcohol use in their military samples [2, 19, 20, 22–25, 27, 29, 30, 33–35, 40, 41]. Hooper *et al.* [29] surveyed 1382 military personnel and reported they had a higher number of units of alcohol consumed per week than the suggested ‘low risk’ drinking threshold. In comparison to the general population, Fear, Iversen [2] identified a greater percentage of hazardous drinkers in a military population than in the general population.

Only two papers considered the potential differences in alcohol consumption between men and women. Fear *et al.* [2] suggested a gender difference in hazardous drinking where there were a greater number of male hazardous drinkers (67% military, 38% general population) than female hazardous drinkers (49% military, 16% general population). However Fadum *et al.* [24] noted that high alcohol consumption did not differ much between military women and men. Furthermore, no significant difference in high alcohol consumption between military and civilian women was found [24].

Papers in this review also considered the consequences of heavy drinking whilst serving in the military. For personnel that had deployed on operational service, a greater volume of drinking was linked to difficulties at home during and post-operational deployment [19, 41]. In addition to demonstrating a high incidence of alcohol use in the military, the papers reported evidence of associations between alcohol use and factors such as age, service type, active deployment, combat exposure, mental health, and relationship status. In UK, US and German military populations, heavy drinking has been associated with holding a lower rank, being younger, being single, being in the Naval service or Army, being deployed to Iraq, not having children, being a smoker, having a combat role and having a parent with a drink or drug problem [2, 19, 23, 25, 35, 41]. Most papers examined such risk factors in soldiers from US or UK military populations, potentially providing limited applicability to military populations in developing nations [22]. Interestingly, in contrast to US and UK findings, Cheng *et al.* [22] identified older age rather than younger age as a significant risk factor for alcohol use in Angola.

Literature suggests that, in the UK, levels of drinking are higher in the Army than other branches of the military [2]. Different subcultures of drinking within individual branches have been attributed to these variations across the military, especially under circumstances that involve personnel taking part in team activities where there may be pressure from peers to drink alcohol to relax and debrief [2]. These situations that involve socialising and alcohol use are a common feature of Armed Forces life and particularly in the Royal Navy. In this regard, Henderson, Langston [27] reported significant degrees of harmful drinking among personnel serving in the Royal Navy in comparison to the civilian population.

In addition to heavy and hazardous drinking, substantial levels of binge drinking have also been found in military populations across UK, US, German, Angolan and Nigerian military populations [2, 20, 22, 25, 30, 33, 40]. Binge drinking in the UK, has been associated with being younger, being in the Army, being single and being a smoker [2]. Furthermore, there appears to be a difference in the prevalence of binge drinking between Active Duty/Regular soldiers and National Guard/Reserves. Larson, Adams [33] identified that there was a marked difference between those personnel in Active Duty who reported frequent binge patterns of alcohol use than those in the National Guard/Reserves.

The issue of problematic alcohol use among the Armed Forces population, related to active service, was considered by 19 papers [6, 19–21, 23, 29–31, 33, 34, 36–44], including a focus on adverse combat experiences [19, 21, 29, 37, 38, 44], pre-deployment preparedness [36] and differences between Active Duty/Regular soldiers and the National Guard/Reserves [31, 33].

Rates of alcohol use have been perceived as the highest for those with combat specific jobs or those with a greater number and higher intensity deployments [6]. Furthermore, despite Special Operations Forces experiencing greater exposure to combat deployments than conventional forces, Dretsch *et al.* [23] determined that the prevalence of alcohol misuse in 16 284 Special Operations Forces soldiers was comparable or lower than reported by the wider military. This paper had the largest sample of the papers included in this review.

Research has indicated that heavy alcohol use often occurs during pre- and post- deployment with a significant association to the deployment period itself [6, 20, 30, 37, 43]. Most of the research primarily focuses on personnel with military service during the Iraq War (2003–2011), with and without deployment experience. More recent papers have begun to consider the impact of the War in Afghanistan (2001–2014).

Adverse combat experiences during deployment have been associated with heavy drinking in military personnel [19, 21, 38]. Hooper et al [29] discussed how the active involvement in theatre of war had strong links to problematic drinking habits among serving personnel who feared for their own mortality and who ‘experienced hostility from civilians’. Wilk, Bliese [44] also found that soldiers who had higher rates of exposure to the threat of death or injury were significantly more likely to screen positive for alcohol misuse. Exposure to atrocities similarly predicted misuse of alcohol with alcohol-related behavioural problems.

Although in Russell [37] all combat experiences were also positively correlated, only the combat experience of killing was significantly related to post-deployment alcohol use. Interestingly, alcohol use decreased amongst those who experienced killing during combat. The authors’ explanation for this was based on the suggestion that the ‘killing experience may activate the soldiers’ mortality salience and trigger a self-preservation focus that manifests itself in reduced risky alcohol consumption’ [37]. No other paper in the review considered this.

Alongside the available evidence that suggests that fighting the enemy in battle and witnessing the horrors of war predisposes serving personnel to the risk of problematic alcohol use, Skipper *et al.* [38] suggested that serving personnel who are part of ‘special forces’ are at an even higher risk of providing a positive alcohol test result due to problematic drinking if they are involved in hostile warfare. In line with adverse combat experiences, Browne, *et al.* [19] also argued that personnel who deployed with their parent unit, whose role in theatre was outside, above or below their training or experience and who experienced poor in-theatre unit leadership were more likely to be heavy drinkers.

Despite several papers in this review (*n* = 13) identifying a significant link between deployment and subsequent alcohol use, regardless of country, Thandi *et al.* [39] demonstrated that levels of drinking were not related to deployment status. Marshall *et al.* [34] also found no effect of deployment on alcohol use, although this was for those who reported pre-deployment depression or PTSD. Three German papers support the suggestion that deployment has no effect on alcohol use [40–42]. Interestingly, the correlates of increased average daily alcohol use across two time points in these papers, were limited social support, greater sleeping difficulties and increased negative post-event cognitions following deployment [41]. Lower PTSD symptom severity pre-deployment and less childhood emotional neglect, predicted a decrease in average daily alcohol consumption [41]. It is possible to suggest that specific deployment experiences impact alcohol use in a military population rather than deployment in general. However, these German papers were all from the same Prevalence, Incidence and Determinant of PTSD and Other Mental Disorders (PID-PTSD^+3^) study. Additionally, Orr *et al.* [36] identified that only pre-deployment preparedness was associated with incident alcohol misuse when controlling for demographics, deployment related factors (e.g. exposure to warzone stressors), and the presence of psychopathology.

US research has identified further differences within a military population. Around 13–14% of National Guard/Reserve personnel exhibited high levels of drinking in association with deployment [31, 33]. Whereas Larson *et al.* [33] discovered that an increase in alcohol use as a result of deployment was actually greater in Active-Duty personnel compared to the National Guard/Reserves. Regardless, alcohol use following deployment is thought to be uniquely predicted by higher levels of PTSD symptom severity, higher levels of avoidance-specific PTSD symptoms and lower levels of positive emotionality [31]. It is worth noting that although there appears to be an indication of differences in alcohol use with engagement type following deployment, more research is needed to further explore this.

Although not a focus of this review, eight papers focussing on alcohol use in a military population reported on the implication of mental health [8, 25, 28, 31, 32, 34, 39, 40]. This included five papers looking at the role and impact of PTSD [25, 31, 32, 34, 39], one on wider mental health [40], one on suicide [28] and one on functional impairment [8].

Previous research has suggested military personnel with PTSD may use alcohol for self-medication as a coping mechanism for distress related to psychological symptoms [31, 34]. Five papers indicated a significant association between PTSD symptom severity, heavy drinking behaviours and new-onset Alcohol Use Disorder [25, 31, 32, 34, 39]. However, once the influence of personality variables were accounted for, Ferrier-Auerbach *et al.* [25] found that mental ill health was not associated with any drinking variable.

Although baseline PTSD symptoms significantly increased the risk of screening positive for new onset alcohol dependence, Kline *et al.* [32] identified no effect of pre-deployment alcohol use on subsequent PTSD diagnoses post-deployment. Such findings indicate that it is possible that the specific psychological consequence of military deployment (e.g. PTSD) significantly impacts military personnel’s alcohol use rather than being in the military in general.

In support of the specific psychological impact of deployment on military personnel’s alcohol use, further differences have been identified between deployed and non-deployed military personnel. Trautmann *et al.* [40] identified among recently deployed soldiers, that heavy drinking was related to a higher risk of anxiety, affective and sleep disorders. Among soldiers never deployed, heavy drinking was linked with any mental disorders other than substance use disorder and was further associated with somatoform disorders. For those recently deployed, associations between heavy drinking and the presence of any mental disorder as well as anxiety disorders were significantly greater than those that had never deployed.

Beyond PTSD, Herberman *et al.* [28] identified that US soldiers who reported high levels of alcohol use were more likely to have seriously considered and/or attempted suicide. After adjusting for level of alcohol use, PTSD, and depression, drinking to avoid rejection/’fit in’ was associated with suicidality.

One paper considered the impact of alcohol use on functional impairment. Rona *et al.* [8] identified that a score on the Alcohol Use Disorder Identification Test (AUDIT) denoting potential alcohol dependence was consistently associated with functional impairment, whereas binge drinking was not. Interestingly, despite a known impairment, participants with hazardous drinking perceived their functioning to be better than those with lower AUDIT scores. The implications of this perception should be explored further. Furthermore, half of the participants presenting with potential alcohol dependence also had psychological comorbidities.

Five papers identified the role of social support and communities in the alcohol consumption of military personnel [6, 22, 26, 30, 39]. The communities within which military personnel reside can have a prominent role in their alcohol use. Besse *et al.* [6] conducted interviews and focus groups with 29 US Active-Duty soldiers to understand the context of alcohol establishments in communities near military installations in relation to alcohol use. Participants identified predatory behaviour by local alcohol establishments to encourage excessive drinking, placing the profit as a higher priority over the safety of the soldiers. Free or reduced admission fees and drink specials were often specifically designed with soldiers in mind, with some participants reporting that alcohol establishments gave soldiers ‘heavier pours’ or mixed drinks with a higher proportion of alcohol. These findings indicate that there is a perception that military personnel drink more, thus increasing the availability of alcohol and subsequent use.

Unsurprisingly, Besse *et al.* [6] also ascertained that drinking has been described as an accepted way to relax and cope with stress brought on by the daily stressors of military life. Drinking alcohol was perceived to be an accepted part of military culture. Pressure to engage in heavy drinking often came from peers as an obligation to prove oneself to the group. This was particularly common among young military personnel or those new to a unit. Socialising appears to play a role in military personnel’s alcohol use. Besse *et al.* [6] conducted the only qualitative paper in this review. The findings allow further insight into the reciprocal relationship between alcohol and military personnel by using interviews and focus groups to explore the reasons behind the relationship. Qualitative methods are highly appropriate for this paper, where the purpose was to learn how military personnel experiences the community within which they reside.

Unexpectedly, Cheng *et al.* [22] indicated that socialising with family and friends two to four times, but not five or more times, per month increased the risk for problematic drinking in military personnel. More specifically, Thandi *et al.* [39] identified that entering into a new relationship resulted in a decrease in alcohol use.

Only two papers considered the role of religion in alcohol consumption [22, 30]. Cheng *et al.* [22] ascertained that attending religious services more than once a week appeared to protect against problematic drinking in Angolan soldiers. It is possible that the effect of religion on alcohol consumption is dependent on the soldiers’ culture as another paper determined that religion had no role in drinking in Nigerian soldiers [30].

The social networks of military members appear to be crucial in the likelihood of alcohol use. For Reserve and National Guard soldiers, one paper indicated that drinking-related social network characteristics such as drinking buddies were associated with increased alcohol problems [26]. However, for those deployed, military-affiliated social networks were a protective factor against alcohol problems [26].

## Discussion

This systematic narrative review explored the relationship between military service and alcohol use. From the 29 papers examined in this review, it is evident that there are military-specific traits and experiences which impact alcohol use, namely military characteristics, such as service type and rank, and military deployment. Mental health, cultural and social factors also play a role in alcohol use in a military population.

Throughout this review, there are associations drawn between military characteristics and alcohol use such as service type, rank and deployment status [2, 19, 23, 25, 35, 41], that have been associated with higher levels of alcohol consumption in military populations in a number of countries [2, 20, 22, 25, 30, 33, 40]. Additionally, studies have pointed towards a difference between alcohol use in a military population and the civilian population [2, 27]. Although, Fadum *et al.* [24] indicated no difference between military women and civilian women.

Most papers indicated that alcohol use was greatest in those with deployment experience, especially those with adverse combat experiences [6, 19, 21, 38]. This is unsurprising, at least in a UK military population, where policy allows the continuation of alcohol use during military ‘decompression’, where those returning from combat receive a short duration of absence together with psychological support [45]. There was a suggestion that these findings were a result of using alcohol as a coping mechanism after traumatic events, however, further work is needed to explore this. Specific combat experiences were significantly related to screening positive for alcohol misuse for elite and non-elite military personnel including personal threats, fighting and atrocities [38]. Interestingly, an increase in alcohol use following deployment was greater for Active-Duty personnel compared to the National Guard and Reserves in a US cohort [33]. It is important to note that not all research found a significant link between deployment and subsequent alcohol use, regardless of country, a few papers argued that deployment had no effect on alcohol use in military populations [34, 39–42].

Evidence in this review suggests that mental ill health and harmful levels of alcohol use in military personnel co-exist. But, more importantly, the evidence suggests that internal stigma makes this population particularly reticent to seek help for both alcohol and mental health problems. Kiernan *et al.* [46] identified that many veterans only present for help when they can no longer cope with the situation, they find themselves in. This study found that seeking help for alcohol misuse issues late, when problems have escalated significantly, invariably led to a co-morbid presentation, a significant decline in mental health and excessive drinking, usually exacerbated by a collapse of the individuals social support network. Once more, this strengthens existing evidence that excess drinking negatively affects physical, social, and mental health [7, 46]. Specifically, PTSD symptom severity was significantly associated with greater alcohol use [25, 31, 32, 34, 39]. Heavy drinking was also related to a higher risk of anxiety, affective and sleep disorders, functional impairment, and suicide [8, 28, 40].

This review also suggests that culture and social factors can influence alcohol use in a military population. An interdependent relationship was identified between military personnel and local alcohol establishments near military installations [6]. It was suggested that establishments tailor their business, often in a predatory way that is perceived as detrimental to military personnel’s health, well-being, and career. The behaviour of alcohol establishments near military installations was felt to exacerbate this alcohol acceptance. However, only one US paper considered this, more international research should be conducted to consider if this is a common experience in a military population. Interestingly, there was a suggestion that attending religious services [22] and that having military affiliated social networks when deployed [26], protected against problematic drinking.

Drinking alcohol was noted as an accepted way to relax and cope with stress in the military with some feeling pressure to conform to drinking [6]. This acceptance of alcohol use and the social norms surrounding its use can have life-long, post-military service implications, as military veterans who normalise or excuse their drinking, delay their engagement in substance misuse services [46]. The historic social and cultural norms within the military [1], and even the ‘romanticising’ of this culture [12] are now being recognised, and the UK’s Ministry of Defence is trying to combat and encourage a sensible approach to alcohol use with initiatives to identify individuals’ alcohol use during regular oral examinations [47].

In almost all studies considered for this review, the Alcohol Use Disorder Identification Test (AUDIT) or the modified brief version, AUDIT-C was utilised. The AUDIT and AUDIT-C have been acknowledged as valid instruments for identifying alcohol misuse or dependence among US and Australian military populations [48, 49]. However, the validity of such tests in other military populations does not appear to have been completed, despite the variability in populations. Regardless of the validity of such instruments, research has relied heavily on self-report questionnaires for assessment in a Military population. This can result in ascertaining large volumes of data; however, this can also give rise to participants answering in a more socially desirable way, rendering results inaccurate [50].

This focus on quantitative research methods has made it difficult to draw any conclusions as to what the resulting impact of military service on alcohol use may have on the ability of service personnel to carry out their jobs. To mitigate this, future research must also consider a qualitative approach, considering personal experiences and meanings ascribed to alcohol use, rather than only symptom severity, to draw any conclusions.

This review has synthesised findings from existing literature, highlighting potential gaps in current research. Despite being a comprehensive, international systematic narrative literature review, it is important to acknowledge there are limitations. First, only papers written in English were considered for review and may have excluded inclusion of international findings. Only the relationship between military service and alcohol use of serving personnel was considered, with papers including veterans in their sample being excluded as it was outside of the aims of this review. Finally, only peer-reviewed research was included in this review and, whilst this was a purposeful decision, it is acknowledged that evidence from grey literature may illuminate further understanding of this issue.

This systematic narrative review aimed to critically evaluate existing literature to explore the relationship between military service and alcohol use. Findings indicated that there are specific characteristics strongly associated with military service that appear to have an impact on alcohol use. The subsequent effects of alcohol use in a military occupational context reinforces the validity of exploring the casual links in more depth. Further research is needed to identify specific ‘risk factors’ associated with serving in the military and problematic alcohol consumption. Exploring the attributable burden will be a crucial building block to developing appropriate and targeted prevention policies.

## References

[1] Jones E , Fear NT. Alcohol use and misuse within the military: a review. Int Rev Psychiatry 2011;23:166–172. 21521086 10.3109/09540261.2010.550868

[2] Fear NT , Iversen A, Meltzer H et al Patterns of drinking in the UK armed forces. Addiction 2007;102:1749–1759. 17935583 10.1111/j.1360-0443.2007.01978.x

[3] London AS , Wilmoth JM, Oliver WJ, Hausauer JA. The influence of military service experiences on current and daily drinking. Subst Use Misuse 2020;55:1288–1299. 32167849 10.1080/10826084.2020.1735438

[4] Irizar P , Leightley D, Stevelink S et al Drinking motivations in UK serving and ex-serving military personnel. Occup Med 2020;70:259–267. 10.1093/occmed/kqaa003 PMC7305700 31961932

[5] Cooper ML , Frone MR, Russell M, Mudar P. Drinking to regulate positive and negative emotions: a motivational model of alcohol use. J Pers Soc Psychol 1995;69:990–1005. 7473043 10.1037//0022-3514.69.5.990

[6] Besse K , Hunt S, Lenk KM. How soldiers perceive the drinking environment in communities near military installations. J Alcohol Drug Educ 2018;62:71–90.

[7] Fear NT , Jones M, Murphy D et al What are the consequences of deployment to Iraq and Afghanistan on the mental health of the UK armed forces? A cohort study. Lancet 2010;375:1783–1797. 20471076 10.1016/S0140-6736(10)60672-1

[8] Rona RJ , Jones M, Fear NT, Hull L, Hotopf M, Wessely S. Alcohol misuse and functional impairment in the UK armed forces: a population-based study. Drug Alcohol Depend 2010;108:37–42. 20047802 10.1016/j.drugalcdep.2009.11.014

[9] Vest BM , Homish DL, Fillo J, Homish GG. Military status and alcohol problems: former soldiers may be at greater risk. Addict Behav 2018;84:139–143. 29679924 10.1016/j.addbeh.2018.04.011 PMC5975126

[10] Mash HBH , Fullerton CS, Ramsawh HJ et al Risk for suicidal behaviors associated with alcohol and energy drink use in the US Army. Soc Psychiatry Psychiatr Epidemiol 2014;49:1379–1387. 24797397 10.1007/s00127-014-0886-0

[11] Namrow N , Rock L. 2012 Workplace and Gender Relations Survey of Reserve Component Members (Survey Note No. 2013-002). Arlington, VA: Defense Manpower Data Center, 2013.

[12] Alcohol Concern. On the Front Line: Alcohol Concern Cymru Briefing. 2012.

[13] Debell F , Fear NT, Head M et al A systematic review of the comorbidity between PTSD and alcohol misuse. Soc Psychiatry Psychiatr Epidemiol 2014;49:1401–1425. 24643298 10.1007/s00127-014-0855-7

[14] Kelsall HL , Wijesinghe MSD, Creamer MC et al Alcohol use and substance use disorders in Gulf War, Afghanistan, and Iraq War veterans compared with nondeployed military personnel. Epidemiol Rev 2015;37:38–54. 25589053 10.1093/epirev/mxu014

[15] Cohen GH , Fink DS, Sampson L, Galea S. Mental health among reserve component military service members and veterans. Epidemiol Rev 2015;37:7–22. 25595172 10.1093/epirev/mxu007 PMC4325668

[16] Snilstveit B , Oliver S, Vojtkova M. Narrative approaches to systematic review and synthesis of evidence for international development policy and practice. J Dev Eff 2012;4:409–429.

[17] Critical Appraisal Skills Programme. *CASP Checklist 2019*. https://casp-uk.net/casp-tools-checklists/.

[18] Braun V , Clarke V. Using thematic analysis in psychology. Qual Res Psychol 2006;3:77–101.

[19] Browne T , Iversen A, Hull L et al How do experiences in Iraq affect alcohol use among male UK armed forces personnel?. Occup Environ Med 2008;65:628–633. 18178589 10.1136/oem.2007.036830

[20] Campbell-Sills L , Ursano RJ, Kessler RC et al Prospective risk factors for post-deployment heavy drinking and alcohol or substance use disorder among US Army soldiers. Psychol Med 2018;48:1624–1633. 29039285 10.1017/S0033291717003105 PMC6620021

[21] Cerdá M , Richards C, Cohen GH et al Civilian stressors associated with alcohol use disorders in the national guard. Am J Prev Med 2014;47:461–466. 25089013 10.1016/j.amepre.2014.06.015 PMC4171186

[22] Cheng KG , Ortiz DJ, Weiss RE et al Patterns of alcohol consumption and factors influencing problematic drinking among Angolan soldiers. J Subst Use 2012;17:138–149.

[23] Dretsch MN , Neff D, Caserta R, Deagle E, Hoge CW, Adler AB. Rates of behavioral health conditions and health risk behaviors in operators and support personnel in US special operations forces. Psychiatry 2020;83:358–374. 32924845 10.1080/00332747.2020.1768787

[24] Fadum EA , Strand LA, Martinussen M, Breidvik L, Isaksen N, Borud E. Fit for fight–self-reported health in military women: a cross-sectional study. BMC Women’s Health 2019;19:1–13. 31623632 10.1186/s12905-019-0820-4 PMC6798407

[25] Ferrier-Auerbach AG , Kehle SM, Erbes CR, Arbisi PA, Thuras P, Polusny MA. Predictors of alcohol use prior to deployment in National Guard Soldiers. Addict Behav 2009;34:625–631. 19375239 10.1016/j.addbeh.2009.03.027

[26] Goodell EMA , Johnson RM, Latkin CA, Homish DL, Homish GG. Risk and protective effects of social networks on alcohol use problems among Army Reserve and National Guard soldiers. Addict Behav 2020;103:106244. 31838442 10.1016/j.addbeh.2019.106244 PMC7045418

[27] Henderson A , Langston V, Greenberg N. Alcohol misuse in the Royal Navy. Occup Med 2009;59:25–31. 10.1093/occmed/kqn152 19074746

[28] Herberman Mash HB , Fullerton CS, Ng THH, Nock MK, Wynn GH, Ursano RJ. Alcohol use and reasons for drinking as risk factors for suicidal behavior in the US Army. Mil Med 2016;181:811–820. 27483518 10.7205/MILMED-D-15-00122

[29] Hooper R , Rona RJ, Jones M, Fear NT, Hull L, Wessely S. Cigarette and alcohol use in the UK armed forces, and their association with combat exposures: a prospective study. Addict Behav 2008;33:1067–1071. 18485610 10.1016/j.addbeh.2008.03.010

[30] Ijomanta IN , Lasebikan VO. Lifetime and 12 months prevalence of alcohol use and alcohol use disorders among soldiers residing in a military community in Ibadan. Subst Use Misuse 2016;51:722–732. 27070029 10.3109/10826084.2016.1153111

[31] Kehle SM , Ferrier-Auerbach AG, Meis LA, Arbisi PA, Erbes CR, Polusny MA. Predictors of postdeployment alcohol use disorders in National Guard soldiers deployed to Operation Iraqi Freedom. Psychol Addict Behav 2012;26:42–50. 21823766 10.1037/a0024663

[32] Kline A , Weiner MD, Ciccone DS, Interian A, Hill LS, Losonczy M. Increased risk of alcohol dependency in a cohort of National Guard troops with PTSD: a longitudinal study. J Psychiatr Res 2014;50:18–25. 24332924 10.1016/j.jpsychires.2013.11.007

[33] Larson MJ , Adams RS, Mohr BA et al Rationale and methods of the Substance Use and Psychological Injury Combat Study (SUPIC): a longitudinal study of army service members returning from deployment in FY2008–2011. Subst Use Misuse 2013;48:863–879. 23869459 10.3109/10826084.2013.794840 PMC3793632

[34] Marshall BDL , Prescott MR, Liberzon I, Tamburrino MB, Calabrese JR, Galea S. Coincident posttraumatic stress disorder and depression predict alcohol abuse during and after deployment among Army National Guard soldiers. Drug Alcohol Depend 2012;124:193–199. 22342428 10.1016/j.drugalcdep.2011.12.027

[35] Mattiko MJ , Olmsted KLR, Brown JM, Bray RM. Alcohol use and negative consequences among active duty military personnel. Addict Behav 2011;36:608–614. 21376475 10.1016/j.addbeh.2011.01.023

[36] Orr MG , Prescott MR, Cohen GH et al Potentially modifiable deployment characteristics and new-onset alcohol abuse or dependence in the US National Guard. Drug Alcohol Depend 2014;142:325–332. 25064024 10.1016/j.drugalcdep.2014.07.005

[37] Russell DW , Russell CA, Riviere LA, Thomas JL, Wilk JE, Bliese PD. Changes in alcohol use after traumatic experiences: the impact of combat on Army National Guardsmen. Drug Alcohol Depend 2014;139:47–52. 24685562 10.1016/j.drugalcdep.2014.03.004

[38] Skipper LD , Forsten RD, Kim EH, Wilk JD, Hoge CW. Relationship of combat experiences and alcohol misuse among US special operations soldiers. Mil Med 2014;179:301–308. 24594465 10.7205/MILMED-D-13-00400

[39] Thandi G , Sundin J, Ng-Knight T et al Alcohol misuse in the United Kingdom armed forces: a longitudinal study. Drug Alcohol Depend 2015;156:78–83. 26409753 10.1016/j.drugalcdep.2015.08.033

[40] Trautmann S , Schönfeld S, Behrendt S, Höfler M, Zimmermann P, Wittchen H. Substance use and substance use disorders in recently deployed and never deployed soldiers. Drug Alcohol Depend 2014;134:128–135. 24210162 10.1016/j.drugalcdep.2013.09.024

[41] Trautmann S , Schönfeld S, Behrendt S et al Predictors of changes in daily alcohol consumption in the aftermath of military deployment. Drug Alcohol Depend 2015;147:175–182. 25499731 10.1016/j.drugalcdep.2014.11.019

[42] Trautmann S , Schönfeld S, Behrendt S et al Stress exposure and the risk for the onset of alcohol use disorders and nicotine dependence in deployed military personnel: the role of prior internalizing disorders. Addict Behav 2015;43:89–96. 25588794 10.1016/j.addbeh.2014.12.013

[43] Vest BM , Heavey SC, Homish DL, Homish GG. Alcohol misuse in reserve soldiers and their partners: cross-spouse effects of deployment and combat exposure. Subst Use Misuse 2018;53:800–807. 29161165 10.1080/10826084.2017.1385632 PMC5951303

[44] Wilk JE , Bliese PD, Kim PY, Thomas JL, McGurk D, Hoge CW. Relationship of combat experiences to alcohol misuse among US soldiers returning from the Iraq war. Drug Alcohol Depend 2010;108:115–121. 20060237 10.1016/j.drugalcdep.2009.12.003

[45] Hacker Hughes JG , Earnshaw NM, Greenberg N et al The use of psychological decompression in military operational environments. Mil Med 2008;173:534–538. 18595415 10.7205/milmed.173.6.534

[46] Kiernan MD , Osborne A, McGill G, Jane Greaves P, Wilson G, Hill M. Are veterans different? Understanding veterans’ help‐seeking behaviour for alcohol problems. Health Soc Care Community 2018;26:725–733. 10.1111/hsc.12585 29851155

[47] MOD. Alcohol Usage in the UK Armed Forces 1 June 2016 – 31 May 2017. 2017.

[48] Crawford EF , Fulton JJ, Swinkels CM, Beckham JC, Calhoun PS. Diagnostic efficiency of the AUDIT-C in U.S. veterans with military service since September 11, 2001. Drug Alcohol Depend 2013;132:101–106. 23465735 10.1016/j.drugalcdep.2013.01.012

[49] Searle AK , Van Hooff M, McFarlane AC et al The validity of military screening for mental health problems: diagnostic accuracy of the PCL, K10 and AUDIT scales in an entire military population. Int J Methods Psychiatr Res 2015;24:32–45. 25511518 10.1002/mpr.1460 PMC6878400

[50] Richman WL , Kiesler S, Weisband S, Drasgow F. A meta-analytic study of social desirability distortion in computer-administered questionnaires, traditional questionnaires, and interviews. J Appl Psychol 1999;84:754–775.

