# Correction to: Military service and alcohol use: a systematic narrative review

**DOI:** 10.1093/occmed/kqac069

**Published:** 2022-07-29

This is a correction to: K Osborne, G Wilson-Menzfeld, G McGill, M D Kiernan, Military service and alcohol use: a systematic narrative review, Occupational Medicine, 2022, kqac045, https://doi.org/10.1093/occmed/kqac045

In the originally published version of this manuscript, there was an error in the Results section of the abstract, which has now been changed to clarify that the role of military affiliated social networks were considered in the study.

This error has been corrected.

